# Assessing social determinants of health needs in newly diagnosed gynecologic oncology patients and their relationship with social support and stress levels

**DOI:** 10.1016/j.gore.2026.102211

**Published:** 2026-09-17

**Authors:** Rose Karvandi, Jennifer Jorgensen, Jeffrey E. Pella

**Affiliations:** a University of Connecticut School of Medicine, UConn Health, Farmington, CT, USA; b Department of Obstetrics and Gynecology, Division of Gynecologic Oncology, UConn Health, Farmington, CT, USA; c Department of Psychiatry, University of Connecticut School of Medicine, Farmington, CT, USA

**Keywords:** Social determinants of health, Gynecologic oncology, Stress, Social support, Cancer screening

## Abstract

**Objective:**

Social determinants of health (SDoH) account for 80% of health outcomes and, with social support and perceived stress, shape treatment experiences and quality of life in gynecologic cancer. This study characterized the prevalence of unmet SDoH needs among patients with newly diagnosed gynecologic cancer and examined associations of SDoH burden with social support and perceived stress.

**Methods:**

Patients with newly diagnosed gynecologic cancer prospectively completed a PRAPARE-based survey assessing 11 SDoH domains, perceived stress, and social support. Logistic regression examined associations of social support and perceived stress with SDoH burden (high burden: ≥ 3 needs).

**Results:**

Fifty-one patients completed screening (68.6% White, 29.4% non-White). Food-related needs were most prevalent (94.1%), followed by public insurance (18.4%) and unemployment (9.8%). Among 49 patients with complete data, 65.3% had one need, 26.5% two, and 8.2% ≥ 3. Most reported high social support (60.8%), and SDoH burden was not associated with social support (OR = 1.00, 95% CI: 0.44–2.24, *p* = .993). Higher perceived stress was associated with increased odds of high SDoH burden (OR = 3.35, 95% CI: 1.17–9.58, *p* = .024). Patients reporting ≥3 needs had higher mean stress (M = 3.25, SD = 1.50) than those with fewer needs (M = 1.53, SD = 1.12).

**Conclusions:**

Unmet SDoH needs were common among patients with newly diagnosed gynecologic cancer. Higher stress was associated with greater SDoH burden, suggesting stress and social needs may co-occur. Screening for SDoH and stress at diagnosis may identify vulnerable patients and guide early interventions.

## Highlights

• All patients with newly diagnosed gynecologic cancer reported at least one unmet social need.
• Food-related need was near-universal (94.1%), likely capturing illness-related barriers as well.
• Higher perceived stress was linked to over three times the odds of high SDoH burden.
• Cumulative SDoH burden was not associated with patients' reported level of social support.
• Findings support routine SDoH and stress screening at gynecologic cancer diagnosis.

## 1 Introduction

Social determinants of health (SDoH) refer to the conditions in which individuals are born, grow, live, work, and age, along with the broader systems and forces that shape everyday life (Hood et al., 2016). The World Health Organization has identified SDoH as major drivers of health inequities, with estimates suggesting that social and environmental factors account for 50–80% of health outcomes, while clinical care contributes approximately 20% (Hood et al., 2016; World Health Organization, n.d.). In oncology, SDoH have emerged as critical yet underexamined contributors to disparities in cancer incidence, treatment access, and survival (Alcaraz et al., 2020). A 2020 policy statement from the American Society of Clinical Oncology (ASCO) formally recognized that cancer disparities and health equity cannot be addressed without attending to social determinants. The statement called for systematic integration of SDoH into cancer care delivery and emphasized that racial and ethnic minority populations bear a disproportionate share of SDoH-related cancer burden (Patel et al., 2020).

Gynecologic cancers represent a significant burden of disease among women in the United States. In 2024, an estimated 117,000 new cases of gynecologic cancers were diagnosed, with approximately 35,000 deaths (American Cancer Society, 2024). Research has increasingly demonstrated that SDoH influence nearly every aspect of the gynecologic cancer experience, from prevention and screening to diagnosis, treatment, and survival (Bristow et al., 2015; Doll, 2018). For example, food insecurity has been associated with delayed care-seeking and poorer nutritional status during treatment (Gundersen and Ziliak, 2015), while transportation barriers can impede access to timely diagnosis and treatment adherence (Syed et al., 2013). Despite this growing body of evidence, routine screening for SDoH in gynecologic oncology settings remains uncommon (Beavis et al., 2020).

Several validated tools exist for systematic SDoH screening. The Protocol for Responding to and Assessing Patients' Assets, Risks, and Experiences (PRAPARE) is a nationally recognized screening instrument developed by the National Association of Community Health Centers to help healthcare organizations identify and address patients' social needs (National Association of Community Health Centers, n.d.). PRAPARE assesses a broad range of domains including housing stability, food access, transportation, education, employment, insurance status, social support, stress, safety, and access to essential resources such as clothing, utilities, and medications. Its structured format makes it well-suited for implementation in clinical settings, and recent work has demonstrated the feasibility of integrating PRAPARE-based tools into electronic health records in primary care. However, its application in oncology populations has been limited (Gold et al., 2018).

Beyond SDoH, psychosocial factors such as social support and perceived stress play important roles in cancer treatment and quality of life. Social support has been shown to buffer the negative effects of cancer diagnosis and treatment, with higher levels of support associated with improved treatment adherence, lower distress, and better survival in some cancer populations (Lutgendorf et al., 2012; Nausheen et al., 2009). Importantly, the relationship between social support and social disadvantage is not straightforward; while patients with greater SDoH burden might be expected to have fewer social resources, some evidence suggests that social support can remain strong even in the setting of significant unmet social needs (Lutgendorf et al., 2012). Understanding whether SDoH burden is associated with social support in gynecologic oncology patients may help clarify whether these factors compound one another or operate independently. Conversely, perceived stress is associated with poorer health behaviors, immune dysregulation, and worse cancer outcomes (Antoni et al., 2006; Schrepf et al., 2013). Stress and unmet social needs may co-occur, as patients facing financial strain, housing instability, or employment challenges may also experience elevated psychological distress. The National Comprehensive Cancer Network (NCCN) recommends distress screening for all cancer patients, yet the association between perceived stress and SDoH burden has not been well characterized in gynecologic oncology specifically (National Comprehensive Cancer Netword, 2024).

A small but growing body of literature has begun to examine SDoH in gynecologic oncology populations. Beavis et al. (2020) demonstrated the feasibility of social resource screening in an urban academic gynecologic oncology clinic. Nyakudarika et al. (2021) found that one-third of patients at an urban public safety-net hospital screened positive for distress and nearly two-thirds had at least one unmet social need, with those factors associated with emergency department visits and treatment interruptions. More recently, the Society of Gynecologic Oncology (SGO) issued a clinical practice statement emphasizing the importance of social needs assessment and intervention in gynecologic oncology care (Lefkowits et al., 2020). However, important gaps remain. Few studies have examined SDoH needs specifically at the time of a new gynecologic cancer diagnosis, when patients may be particularly vulnerable, and even fewer have simultaneously assessed the interplay among SDoH burden, perceived stress, and social support.

The purpose of the present study was to address these gaps by: (1) characterizing the prevalence of unmet SDoH needs among patients with newly diagnosed gynecologic cancers at an academic medical center; (2) examining the association between cumulative SDoH burden and social support level; and (3) examining the association between perceived stress and SDoH burden. We hypothesized that patients with newly diagnosed gynecologic cancers would have substantial unmet SDoH needs, and that higher perceived stress would be associated with greater SDoH burden.

## 2 Methods

### 2.1 Study design and setting

This was a prospective, cross-sectional observational study conducted at UConn Health, an academic medical center in Farmington, Connecticut, USA. The study was determined to be exempt by the UConn Health Institutional Review Board. All patients were recruited from the Division of Gynecologic Oncology between September 2023 and September 2025.

### 2.2 Participants

Eligible participants were adult patients (aged 18 years or older) with a new diagnosis of gynecologic cancer (uterine, ovarian, cervical, vaginal, or vulvar) presenting to the gynecologic oncology clinic at UConn Health. Patients were approached in the clinical setting after their initial treatment-planning visits. Patients who were unable to provide informed consent or who did not speak English were excluded. All participants provided written informed consent prior to enrollment.

### 2.3 Measures

*Social Determinants of Health.* SDoH needs were assessed using a survey adapted from the PRAPARE tool (National Association of Community Health Centers, n.d.). The adapted survey was administered by trained research volunteers in the clinic setting. Eleven SDoH domains were assessed: (1) food-related need, (2) clothing, (3) utilities, (4) phone access, (5) medication access, and (6) childcare, which were derived from a single multi-select PRAPARE item asking patients whether they or a family member had been unable to obtain any of these resources when needed in the past year; (7) housing instability, assessed via items on current housing status and concern about housing loss; (8) transportation barriers, assessed via an item on whether lack of transportation had prevented attendance at medical appointments; (9) less than a high school degree; (10) unemployment, defined as currently unemployed and seeking work; and (11) public insurance, defined as having Medicaid as the primary insurance type. Each domain was scored as an unmet need (present) or no unmet need (absent). Cumulative SDoH burden was calculated as the total number of domains endorsed. High SDoH burden was defined as ≥3 unmet needs for analytic purposes.

*Note on the food-related need item.* The PRAPARE food item asks whether the patient or a family member has been unable to obtain food when needed. This item appears to have captured a broader construct than traditional food insecurity as measured by validated instruments such as the USDA Household Food Security Survey. In a newly diagnosed cancer population, endorsement may reflect illness-related barriers (e.g., appetite loss, nausea, physical difficulty shopping) in addition to socioeconomic barriers. The high prevalence observed in this sample (94.1%) should be interpreted in light of this broader construct, and we use the term “food-related need” rather than “food insecurity” throughout.

*Perceived Stress.* Perceived stress was assessed using the PRAPARE stress item, which asks respondents to rate their current level of stress (“Stress is when someone feels tense, nervous, anxious, or can't sleep at night because their mind is troubled. How stressed are you?”). Responses were recorded on a 5-point scale: 0 = “Not at all,” 1 = “A little bit,” 2 = “Somewhat,” 3 = “Quite a bit,” and 4 = “Very much.” Stress was entered as a continuous variable in regression analyses to maximize statistical power given the sample size.

*Social Support.* Social support was assessed using the PRAPARE social integration item, which asks respondents how often they see or talk to people they care about and feel close to (e.g., talking to friends on the phone, visiting friends or family, going to church or club meetings). Response options were: “Less than once a week,” “1 or 2 times a week,” “3 to 5 times a week,” and “5 or more times a week.” For analysis, responses were dichotomized into low social support (fewer than 5 times per week) and high social support (5 or more times per week).

*Relationship of the single-item measures to validated instruments.* Perceived stress and social support were each assessed with a single PRAPARE item rather than a validated multi-item scale. Single-item stress measures have demonstrated satisfactory criterion and construct validity for group-level analysis and correlate with the Perceived Stress Scale (PSS) and other multi-item instruments (*r* = 0.31 to 0.46), a range comparable to the correlations observed among those instruments themselves (Elo et al., 2003; Cohen et al., 1983; Littman et al., 2006). The PRAPARE social support item indexes frequency of contact and therefore measures structural support, whereas instruments such as the Medical Outcomes Study Social Support Survey (MOS-SSS) assess the perceived adequacy and functional dimensions of support (Sherbourne and Stewart, 1991). Both items were retained because they were embedded in the screening instrument already administered in the clinic, and the resulting trade-off in measurement precision is considered in the Discussion.

*Demographic Variables.* Race and ethnicity were collected. Race was categorized as White or non-White for descriptive purposes given the sample size.

### 2.4 Statistical analysis

Descriptive statistics were used to characterize the sample and the prevalence of individual SDoH needs. Frequencies and percentages were calculated for categorical variables; means and standard deviations were reported for continuous variables. A binary logistic regression tested whether cumulative SDoH burden (continuous count of unmet needs) was associated with social support level (high vs. low). A second binary logistic regression tested whether perceived stress (continuous, 0–4) was associated with high SDoH burden (≥ 3 unmet needs). Race was examined as a potential covariate given well-documented racial disparities in SDoH burden among cancer populations (Patel et al., 2020); however, race was not significantly associated with either outcome variable and produced quasi-complete separation in the model predicting high SDoH burden due to the small number of events (*n* = 4), resulting in unstable coefficient estimates. Race was therefore not retained in the final models. Model fit was assessed using the Nagelkerke R^2^ statistic. Unstandardized coefficients (B) with standard errors (SE) and odds ratios (OR) with 95% confidence intervals were reported. Supplementary nonparametric analyses (Mann-Whitney U, Fisher's exact test) were conducted to corroborate the stress result given the small event count. A two-tailed *p*-value of <0.05 was considered statistically significant. Patients with incomplete data on analytic variables were excluded using listwise deletion, resulting in *N* = 49 for the social support model and *N* = 48 for the perceived stress model (one additional patient declined to answer the stress item). Analyses were conducted using SPSS version 31.

## 3 Results

### 3.1 Sample characteristics

A total of 51 patients with newly diagnosed gynecologic cancers completed the PRAPARE-based screening survey. The majority identified as White (*n* = 35, 68.6%), with 15 patients (29.4%) identifying as non-White and one declining to report race. Six patients (11.8%) identified as Hispanic or Latino. Most spoke English as their primary language (*n* = 45, 88.2%). Thirty two patients (62.7%) had completed more than a high school education. Over half held private insurance (*n* = 26, 53.1%), followed by Medicare (*n* = 10, 20.4%) and Medicaid (*n* = 6, 12.2%). With respect to employment, 22 patients (43.1%) were not seeking work (e.g., retired, disabled, student), 17 (33.3%) were employed full-time, 7 (13.7%) were employed part-time or in temporary work, and 5 (9.8%) were unemployed. The mean perceived stress score was 1.62 (SD = 1.23) on a 0–4 scale. Complete sample characteristics are presented in Table 1.

**Table 1:** Sample Characteristics (*N* = 51).

Characteristic	n	%
**Race**		
White	35	68.6
Non-White	15	29.4
Not reported	1	2.0
**Ethnicity**		
Hispanic/Latino	6	11.8
Non-Hispanic/Latino	45	88.2
**Primary Language**		
English	45	88.2
Spanish	5	9.8
Other	1	2.0
**Education**		
More than high school	32	62.7
High school diploma or GED	17	33.3
Less than high school	2	3.9
**Insurance**		
Private	26	51.0
Medicare	10	19.6
Medicaid	6	11.8
Private + Medicare	4	7.8
None/uninsured	2	3.9
Medicare + Medicaid	1	2.0
Not reported	1	2.0
**Employment Status**		
Not seeking work (retired, disabled, student)	22	43.1
Full-time	17	33.3
Part-time or temporary	7	13.7
Unemployed	5	9.8
**Social Support**		
High (≥5 times/week)	31	60.8
Low (<5 times/week)	20	39.2
**Perceived Stress (*N* = 50)**		
Mean (SD)	1.62 (1.23)	
Not at all	8	16.0
A little bit	20	40.0
Somewhat	11	22.0
Quite a bit	5	10.0
Very much	6	12.0

Note. Stress scored on a 0–4 scale (0 = Not at all, 4 = Very much); N = 50 due to one patient declining to answer. Social support defined as high if patient reported seeing/talking to close others ≥5 times per week.

### 3.2 Prevalence of unmet SDoH needs

All 11 SDoH domains were assessed across the full sample. Food-related need was the most prevalent, endorsed by 48 of 51 patients (94.1%). As noted in the Measures section, this item captures a broad construct that may include illness-related barriers to food access in addition to socioeconomic food insecurity. Beyond the food item, the most common unmet needs were public insurance as primary coverage (Medicaid; *n* = 9, 18.4%), unemployment (*n* = 5, 9.8%), housing instability (*n* = 4, 7.8%), and transportation barriers (*n* = 3, 5.9%). Two patients (3.9%) had less than a high school education, one reported difficulty accessing clothing (2.0%), and one reported difficulty affording medications (2.0%). No patients endorsed unmet needs for utilities, phone access, or childcare. The prevalence of individual SDoH domains is presented in Table 2.

**Table 2:** Prevalence of Individual Unmet SDoH Needs (*N* = 51).

SDoH Domain	n/N	%
Food-related need	48/51	94.1
Public insurance (Medicaid)	9/49	18.4
Unemployment	5/51	9.8
Housing instability	4/51	7.8
Transportation barriers	3/51	5.9
Less than HS education	2/51	3.9
Clothing	1/51	2.0
Medication access	1/51	2.0
Utilities	0/51	0.0
Phone access	0/51	0.0
Childcare	0/51	0.0

Note. SDoH = social determinants of health. Food-related need reflects a broad PRAPARE item that may capture illness-related barriers in addition to socioeconomic food insecurity (see Measures). Unemployment defined as currently unemployed and seeking work. Public insurance defined as Medicaid as primary coverage (*N* = 49 due to missing data). All other domains *N* = 51.

Among the 49 patients with complete SDoH data across all 11 domains, 32 (65.3%) had one unmet need, 13 (26.5%) had two unmet needs, and 4 (8.2%) had three or more unmet needs. The mean number of unmet SDoH needs was 1.45 (SD = 0.71). All patients in the analytic sample endorsed at least one unmet SDoH need.

### 3.3 Social support and SDoH burden

The majority of patients reported high social support, defined as seeing or talking to close others five or more times per week (*n* = 31, 60.8%); 20 patients (39.2%) reported low social support. A binary logistic regression was conducted to examine the association between cumulative SDoH burden (continuous count) and social support level. SDoH burden was not significantly associated with social support (B = −0.004, SE = 0.41, OR = 1.00, 95% CI: 0.44–2.24, *p* = .993). The overall model was not statistically significant (χ^2^(1) = 0.00, *p* = .993, Nagelkerke R^2^ < 0.001, *N* = 49).

### 3.4 Perceived stress and SDoH burden

A binary logistic regression was conducted to examine the association between perceived stress (continuous, 0–4) and high SDoH burden (≥ 3 unmet needs). Higher perceived stress was significantly associated with increased odds of high SDoH burden (B = 1.21, SE = 0.54, OR = 3.35, 95% CI: 1.17–9.58, *p* = .024). The model was statistically significant (χ^2^(1) = 7.10, *p* = .008, Nagelkerke R^2^ = 0.32, *N* = 48). Patients with ≥3 unmet needs reported markedly higher mean stress (M = 3.25, SD = 1.50) compared to those with fewer than three needs (M = 1.53, SD = 1.12).

Given the small number of patients with high SDoH burden (*n* = 4), supplementary nonparametric analyses were conducted to corroborate the logistic regression finding. A Mann-Whitney *U* test confirmed that perceived stress scores were significantly higher among patients with ≥3 needs than among those with fewer needs (U = 32.5, *p* = .033). Fisher's exact test comparing high stress (scores of 3–4) versus low stress (scores of 0–2) by SDoH burden group was also significant (OR = 13.50, *p* = .033). The point-biserial correlation between perceived stress and high SDoH burden was *r* = 0.39 (*p* = .006), representing a medium-to-large effect.

## 4 Discussion

This study examined unmet SDoH needs, social support, and perceived stress among patients with newly diagnosed gynecologic cancer. All patients in the analytic sample reported at least one unmet SDoH need, underscoring the widespread presence of social needs at the time of diagnosis. This near-universal prevalence exceeds the roughly two-thirds rate reported in a prior gynecologic oncology safety-net population (Nyakudarika et al., 2021) and was driven largely by the food-related item, endorsed by 94.1% of participants. While food insecurity is frequently identified as a concern among oncology patients (Gundersen and Ziliak, 2015), the high prevalence observed likely reflects the broad wording of the PRAPARE food item, which asks whether patients or family members were unable to obtain food when needed. In the context of a new cancer diagnosis, endorsement may have captured illness-related barriers such as nausea, appetite loss, or difficulty shopping for and preparing meals, in addition to socioeconomic food insecurity. Future studies should pair the PRAPARE food item with a validated food insecurity measure, such as the USDA two-item screener, to better distinguish socioeconomic food insecurity from cancer-related barriers to food access and to clarify the prevalence of each within this population.

A second key finding was the significant association between perceived stress and SDoH burden. Patients reporting higher stress had more than three times the odds of high SDoH burden (≥ 3 unmet needs; OR = 3.35), an association that was robust across logistic regression, nonparametric testing, and correlation analyses. Although the cross-sectional design precludes causal inference, the relationship between stress and unmet social needs is likely bidirectional. Patients facing housing instability, financial strain, transportation barriers, or other social challenges may experience heightened psychological distress while navigating a new cancer diagnosis and treatment. The elevated stress may in turn impair their ability to access resources and coordinate care, further compounding unmet needs. These findings indicate that stress and social needs co-occur in gynecologic oncology patients and align with NCCN recommendations for routine distress screening (National Comprehensive Cancer Network, 2024). Clinically, patients with elevated stress at diagnosis may warrant enhanced assessment of unmet social needs and timely referral to supportive services.

In contrast, SDoH burden was not associated with social support level, suggesting that social support may operate independently of social vulnerability in this population. Patients with multiple unmet needs may still maintain strong social networks, with family and friends providing emotional support regardless of unmet social needs, reflecting the role of resilience during cancer treatment. This pattern is consistent with prior work showing that social support influences cancer outcomes through pathways that are not reducible to socioeconomic resources (Lutgendorf et al., 2012). The null association may reflect measurement limitations: social support was assessed with a single PRAPARE item capturing frequency of social contact, which may not reflect the perceived adequacy of support. More comprehensive measures may reveal associations not detected here.

Several limitations should be considered when interpreting these findings. The sample was modest (*N* = 51), with only four patients meeting criteria for high SDoH burden, limiting statistical power for the perceived stress model. The small number of patients with high SDoH burden also prevented reliable adjustment for race in the regression analyses. The study was conducted at a single academic medical center and excluded patients who did not speak English, both of which limit generalizability, particularly given the equity focus of SDoH research. The cross-sectional design does not permit causal determinations among SDoH burden, stress, and social support. Perceived stress and social support were each assessed with single-item PRAPARE questions rather than validated multi-item instruments such as the Perceived Stress Scale (PSS) (Cohen et al., 1983) or the Medical Outcomes Study Social Support Survey (MOS-SSS) (Sherbourne and Stewart, 1991). Although single-item stress measures correlate with validated multi-item scales at levels comparable to those observed among the scales themselves (Elo et al., 2003; Littman et al., 2006), they are less reliable for classifying individual patients, and the social support item captures frequency of contact rather than perceived adequacy of support. Both forms of measurement error would be expected to attenuate rather than inflate the associations tested here, so the null social support finding should be read as inconclusive rather than as evidence against an association. The sample was also predominantly White, limiting the ability to examine racial and ethnic differences. Finally, as noted, the PRAPARE food item likely captured a broader construct than traditional food insecurity and may have overestimated the prevalence of food-related social need.

Despite these limitations, the study has several clinical implications. The near-universal prevalence of at least one unmet need supports routine SDoH screening at the time of gynecologic cancer diagnosis, when early identification of social barriers can facilitate referrals to social work, financial assistance, transportation services, and other community resources. The volunteer-administered PRAPARE-based survey proved feasible in this setting, demonstrating that SDoH screening can be incorporated into gynecologic oncology practice. Moreover, the association between perceived stress and SDoH burden suggests that a brief stress screen may help triage patients for more comprehensive social needs assessment. Taken together, these findings support integrating SDoH and psychosocial screening into routine oncology care to identify socially vulnerable patients early and promote patient-centered care.

## CRediT authorship contribution statement

**Rose Karvandi:** Writing – original draft, Investigation, Formal analysis, Data curation, Conceptualization. **Jennifer Jorgensen:** Conceptualization, Supervision. **Jeffrey E. Pella:** Methodology, Formal analysis, Supervision, Writing – review & editing.

## Ethics declaration

Written informed consent to take part in the study and to publish the article has been obtained from all participants or their legal representatives. The privacy rights of participants have been observed.

Written informed consent was obtained from all individual participants included in the study.

This study was reviewed by the University of Connecticut Health Institutional Review Board and determined to be exempt.

This study was performed in compliance with relevant laws, regulatory frameworks and guidelines where the research took place.

Ethics committee approval was not required under relevant laws and institutional guidelines. The UConn Health Institutional Review Board determined this study to be exempt human subjects research (IRB Number 24×-016-2; exempt determination November 14, 2023) under 45 CFR 46.104(d)(2) and 46.104(d)(4)(iii). Formal ethics approval was not required. The study was conducted in accordance with 45 CFR 46, the HIPAA Privacy Rule, the Declaration of Helsinki, and UConn Health institutional policy. All participants were adults and provided written informed consent prior to enrollment.

## Funding

This work was supported by the Summer Research Program at the University of Connecticut School of Medicine. The funding source had no role in study design; in the collection, analysis, or interpretation of data; in the writing of the report; or in the decision to submit the article for publication.

## Declaration of competing interest

The authors declare that they have no known competing financial interests or personal relationships that could have appeared to influence the work reported in this paper.

## Data Availability

The data that support the findings of this study are available from the corresponding author upon reasonable request.
